# 2249. Piperacillin-tazobactam-targeted provider education to improve utilization in a small community hospital

**DOI:** 10.1093/ofid/ofad500.1871

**Published:** 2023-11-27

**Authors:** Robert Gilmore, Jennifer Elgin, Cirle A Warren

**Affiliations:** UVA Community Health, Culpepper, Virginia; UVA Community Health, Culpepper, Virginia; University of Virginia, Charlottesville, Virginia

## Abstract

**Background:**

Piperacillin-tazobactam is a broad-spectrum antibiotic commonly used for the treatment of *Pseudomonas aeruginosa* and other susceptible Gram negative and anaerobic infections. Piperacillin-tazobactam utilization is markedly elevated in our institution compared with regional and national rates.Thus, interventions to promote optimal use of this antibiotic to reduce utilization rates and negative consequences are needed.

**Methods:**

Healthcare providers were provided with guidelines on piperacillin-tazobactam alternative treatments for various infections. Guideline handouts were disseminated and complemented by in-person presentations and one-on-one case discussions, as needed. Data on piperacillin-tazobactam utilization was gathered using BD Medmined’s medication analytics.

**Results:**

Total antibiotic utilization at our institution is higher compared to national and regional rates. In the 2^nd^ quarter (Q2) of 2021, piperacillin-tazobactam utilization was 44% and 26% above national and regional comparative rates, respectively. In Q2 2022, piperacillin-tazobactam was 59% and 45% higher, respectively. Immediately post-initiation of intervention (Q3 2022), utilization rates dropped to 34% and 22%, respectively and utilization dropped further in the succeeding quarters attaining comparable rates to national and regional comparator hospitals.

**Conclusion:**

Providing healthcare providers with antibiotic-targeted education on alternative treatments can significantly reduce the utilization of piperacillin-tazobactam. Further studies are warranted to evaluate the long-term impact of this intervention on antibiotic stewardship in small community hospitals.

**Disclosures:**

**Cirle A. Warren, MD**, Ser-109 Coactuate: Advisor/Consultant|Ser-109 Coactuate: Honoraria

